# Comparative clinical outcomes of robot-assisted liver resection versus laparoscopic liver resection: A meta-analysis

**DOI:** 10.1371/journal.pone.0240593

**Published:** 2020-10-13

**Authors:** Lilong Zhang, Qihang Yuan, Yao Xu, Weixing Wang

**Affiliations:** 1 Department of Hepatobiliary and Laparoscopic Surgery, Renmin Hospital of Wuhan University, Wuhan, Hubei, China; 2 Department of General Surgery, The First Affiliated Hospital of Dalian Medical University, Dalian, Liaoning, China; 3 Surgical Intensive Care Unit (SICU), Department of General Surgery, Jinling Hospital, Medical School of Nanjing University, Nanjing, Jiangsu, China; International Medical University, MALAYSIA

## Abstract

**Background:**

As an emerging technology, robot-assisted surgical system has some potential merits in many complicated endoscopic procedures compared with laparoscopic surgery. But robot-assisted liver resection is still a controversial problem on its advantages compared with laparoscopic liver resection. We aimed to perform the meta-analysis to assess and compare the clinical outcomes of robot-assisted and laparoscopic liver resection.

**Methods:**

We searched PubMed, Cochrane Library, Embase databases, Clinicaltrials, and Opengrey through March 24, 2020, including references of qualifying articles. English-language, original investigations in humans about robot-assisted and laparoscopic hepatectomy were included. Titles, abstracts, and articles were reviewed by at least 2 independent readers. Continuous and dichotomous variables were compared by the weighted mean difference (WMD) and odds ratio (OR), respectively.

**Results:**

Of 936 titles identified in our original search, 28 articles met our criteria, involving 3544 patients. Compared with laparoscopy, the robot-assisted groups had longer operative time (WMD: 36.93; 95% CI, 19.74–54.12; P < 0.001), lower conversion rate (OR: 0.63; 95% CI, 0.46–0.87; P = 0.005), higher transfusion rate (WMD: 2.39; 95% CI, 1.51–3.76; P < 0.001) and higher total cost (WMD:0.49; 95% CI, 0.42–0.55; P < 0.001). In addition, the baseline characteristics of patients about largest tumor size was larger (WMD: 0.36; 95% CI, 0.16–0.56; P < 0.001) and malignant lesions rate was higher (WMD: 1.50; 95% CI, 1.21–1.86; P < 0.001) in the robot-assisted versus laparoscopic hepatectomy. The subgroup analysis of minor hepatectomy showed robot-assisted was associated with longer operative time (WMD: 36.00; 95% CI, 12.59–59.41; P = 0.003), longer length of stay (WMD: 0.51; 95% CI, 0.02–1.01; p = 0.04) and higher total cost (WMD: 0.48; 95% CI, 0.25–0.72; P < 0.001) (Table 3); while the subgroup analysis of major hepatectomy showed robot-assisted was associated with lower estimated blood loss (WMD: -122.43; 95% CI, -151.78–-93.08; P < 0.001).

**Conclusions:**

Our meta-analysis revealed that robot-assisted was associated with longer operative time, lower conversion rate, higher transfusion rate and total cost, and robot-assisted has certain advantages in major hepatectomy compared with laparoscopic hepatectomy.

## Introduction

Liver resection is deemed to be one of the most effective treatments for liver neoplasms. Since Reich et al. reported the first laparoscopic liver resection (LLR) in 1991, LLR has been widely used in the treatment of various liver diseases [1]. Many non-randomized studies showed that LLR was safe and effective for the treatment of liver tumors when compared to open liver resection [2–4]. However, the merits of LLR were downplayed because of the limited operating space, difficulties of exposure, the complicacy of the bile duct and hepatic vascular structures, and intraoperative bleeding tendencies [5,6]. Besides, the technique of laparoscopic hepatectomy has its defects which are difficult to overcome, such as the lack of depth perception, the inevitable hand tremor, the surgeon fatigue after the lengthy surgery and the limitation by a fixed pivot point with only a two-dimensional view and four-degree of freedom [7].

The robot-assisted surgery system has overcome some technical bottlenecks of laparoscopy and greatly improves the flexibility and precision of hepatectomy operation [8,9]. It can also provide the magnified field of surgery and the three-dimensional view, which facilitates intracorporeal sutures and delicate tissue anatomy [10]. However, several limitations impede its popularization, such as high cost and high machine failure rate.

Although there were meta-analyses as regards RLR versus LLR for liver tumors, the articles only included studies published before 2017 and, most importantly, their findings remained controversial [11–14]. For example, Guan et al. [13] revealed that, compared with LLR, the RLR had higher estimated blood loss; while Qiu et al. [12] found that there were no significant differences between the two groups in blood loss. Interestingly, we found almost ten new published studies related to compare the outcomes of RLR versus LLR in treatment for liver neoplasms and several related studies published before 2017 which were not included in previous meta-analyses. Thus, we aimed to perform an updated meta-analysis to assess and compare the clinical outcomes of RLR and LLR comprehensively.

## Methods

The meta-analysis was conducted according to the Meta-Analysis of Observational Studies in Epidemiology recommendations and the Preferred Reporting Items for Systematic Reviews and Meta-Analyses guidelines [15,16].

### Literature search strategy

We searched electronic databases of PubMed, Embase, the Cochrane Library, Clinicaltrials (https://clinicaltrials.gov/), and Opengrey (http://www.opengrey.eu/) on March 24, 2020, restricted to the English language. The following terms were searched in [Title/Abstract]: robotics [MeSH], robot*, Da Vinci, telerobotics, computer-assisted, laparoscopy [MeSH], laparoscop*, coelioscop*, celioscop*, peritoneoscop*, hepatectomy [MeSH], hepatic resection, liver resection, liver surgery. In addition, studies were manually searched in the reference lists of all retrieved articles.

### Study selection criteria

Two authors (Lilong Zhang, Qihang Yuan) independently screened the literature, according to the inclusion criteria and exclusion criteria, and all disagreements were dealt with by the senior authors (Weixing Wang). Inclusion criteria: (1) intervention: RLR versus LLR, including hand-assisted LLR; (2) reporting at least one of the clinical outcomes of interest, which will be explained in detail later; (3) if multiple studies of the same population were reported, only kept the most recent or complete article. Exclusion criteria: (1) Hepatectomies for living donor transplantation were excepted; (2) Case reports, comments, review articles, editorials, letters to the editor, and experimental animal studies were excluded.

### Data extraction

Two authors (Lilong Zhang, Qihang Yuan) independently extracted and summarized the following parameters from each study: the first author, year of publication, country, study design, No. of patients, types of hepatectomy, age, gender, body mass index (BMI) and the clinical outcomes of interest, including (1) intraoperative outcome indicators: operative time (minutes), estimated blood loss (mL), transfusion rate, conversion rate; postoperative outcome indicators: overall surgical complications, minor surgical complications, major surgical complications, length of hospital stay (days), mortality rate; pathological outcome indicators: R0 resection rate, R1 resection rate, malignant lesions rate, largest tumor size (cm) and total cost ($). Furthermore, if there was inaccurate or missing information extracted from the original article, we attempted to contact the corresponding authors of studies to guarantee data accuracy.

### Quality assessment

Two authors (Lilong Zhang, Qihang Yuan) independently assessed the methodological quality of the included articles. The Newcastle-Ottawa Scale (NOS), a risk assessment tool recommended by the Cochrane collaboration, was applied to evaluate the quality of non-randomized controlled studies. The following factors were taken into account: patient selection, comparability of the study groups, and assessment of outcome. The maximum score obtained by this scoring system was 9, and studies with scores ≥7 were defined as high quality [16].

### Statistical analysis

The meta-analyses were performed by using Review Manager V.5.3 and Stata 15.0 (metan package). Continuous and dichotomous variables were compared by the weighted mean difference (WMD) and odds ratio (OR), respectively. For studies that presented continuous data as median and range values or median and interquartile, the means and standard deviations were calculated using statistical algorithms described by Luo et al. [17] and Wan et al. [18], respectively. All the effect quantities were represented by 95% confidence interval (CI). P<0.05 was considered statistically significant. Statistical heterogeneity between studies was assessed by I^2^ statistic was used to quantify the heterogeneity. If I^2^ > 50%, indicating statistically significant heterogeneity between studies, the random-effect model was used; otherwise, the fixed-effect model was used [19].

Sensitivity analyses were conducted in the studies of retrospective non-matched and retrospective matched comparative studies. Potential publication bias was evaluated using the Begg, Egger tests, and funnel plot [20,21]. According to the Cochrane handbook, when more than 10 studies were included in an outcome indicator, funnel plots could be used to reflect the publication bias; otherwise, the publication bias was difficult to be expressed by funnel plots, and funnel plot analysis could be omitted [22].

## Results

### Study selection and study characteristics

**Fig 1:**
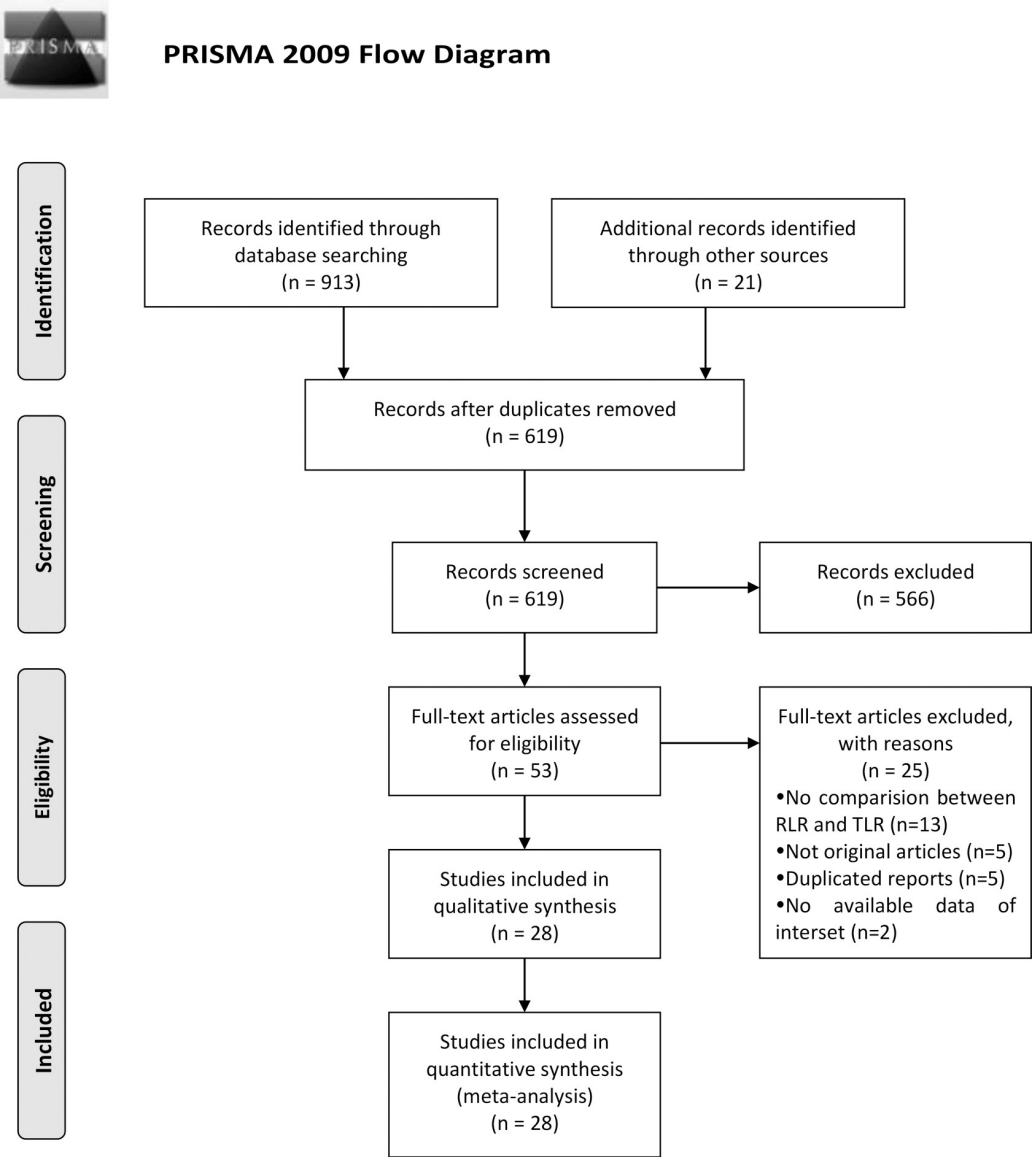
Flow diagram of studies identified, included, and excluded.

In this meta-analyses, identified 934 potentially eligible records, and screened the titles and abstracts of these records for inclusion. On examination of the full text of 53 records, and 28 [23–50] met our inclusion criteria (Fig 1), including 3544 cases (1312 cases for RLR and 2232 cases for LLR). Although the studies of Chen et al. [51] and Kim et al. [52] both met the research topics, they were excluded from the meta-analysis because the articles of them only provided abstract and, more importantly, we were unable to get data of interest. Among the 28 studies included in the final meta-analysis, 7 were from China, 7 from the United States, 4 from Korea, 3 from Italy, 2 from France,1 from Germany, 1 from Russian, 1 from Poland, 1 from Belgium and 1 from France and Italy. The characteristics of the included articles were shown in Table 1.

**Table 1:** Characteristics of the included studies.

First author, year of publication,Country	Study design	No. of patients		Types of hepatectomy	Age, RAH/LLR, mean or median	Gender, RLR/LLR, No.of males	BMI (kg/m2), mean or median	Largest tumor size (cm), RLR/LLR, mean or median	Quality scores	Operation indications
		RLR	LLR							
Al-Temim,2019,USA	M	123	123	Major and minor	Unclear	Unclear	Unclear	Unclear	5	3,6,8,9
Berber,2010,USA	P	9	23	Minor	66.6/66.7	7/12	Unclear	3.2±1.3/2.9±1.3	8	1,2,3,8,12,14
Chong,2019,China	P	91	92	Major and minor	58.7/59.8	65/60	24.6/23.5	Unclear	8	1,2,3,4,5,7,8,10,14
Cortolillo,2018,USA	R	204	520	Major and minor	57.5/60.1	Unclear	Unclear	Unclear	6	7,9
Croner,2016,Germany	P	10	19	Minor	64.0/59.0	8/13	28.0/26.0	5.59±2.46/4.42±1.82	7	1,3,4,5,7,9,10,11,12,14
Efanov,2016,Russian	R	16	35	Major and minor	Unclear	Unclear	Unclear	Unclear	5	8
Fruscione,2019,USA	R	57	116	Major	58.1/53.2	20/52	28.1/29.5	Unclear	7	1,2,3,4,5,7,14
Hu,2019,China	R	58	54	Minor	52.2/48.9	26/33	24.7/23.8	4.7 ± 2.6/4.7 ± 2.8	7	1,2,6,7,8,9,10,11,12,13,14
Ji,2011,China	R	13	20	Major and minor	Unclear	Unclear	Unclear	Unclear	7	1,3,4,5,6,8,11
Kim,2016,Korea	R	12	31	Minor	54.1/56.4	6/18	Unclear	2.67±1.34/2.36±1.01	8	1,2,3,6,7,9,11,12,13,14
Lai,2016,China	P	100	35	Major and minor	62.1/57.9	66/26	Unclear	3.3±1.9/2.7±1.3	7	1,2,3,6,7,8,9,10,12,14
Lee KF,2015,China	R	70	66	Major and minor	58.0/58.0	46/39	Unclear	3.06±2.32/2.84±1.79	8	1,2,3,6,7,8,9,11,12,14
Lee SJ,2019,Korea	R	13	10	Major and minor	62.2/58.8	7/5	24.6/23.5	4.13±2.38/3.28±1.80	7	2,3,4,5,6,7,8,9,12,14
Lim,2019,FI	P	61	111	Major and minor	66/63	41/83	25/26	4.4±2.8/3.3 ±2.3	8	1,3,4,5,6,7,9,11,12,14
Magistr,2017,Italy	R	22	24	Major and minor	60.9/66.6	18/15	26.8/26.5	3.40±1.35/2.26±1.13	8	1,2,3,4,5,6,7,8,9,10,11,12
Marino,2018,Poland	R	14	20	Major	58.3/62.3	8/11	28.2/27.9	4.51±0.51/4.48±0.81	7	1,2,3,6,7,8,9,10,11,12
Mejia,2019(A),USA	R	35	85	Minor	65.0/55.0	16/36	27.0/27.6	4.46±3.48/3.73±2.64	8	1,2,3,4,5,6,7,8,9,10,12,13,14
Mejia,2019(B),USA	R	8	13	Major	62.0/47.0	4/6	28.6/29.1	6.91±4.38/5.99±3.90	8	1,2,3,4,5,6,7,9,10,13,12,14
Montalti,2015,Italy	M	36	72	Minor	62.0/56.8	21/39	Unclear	4.44±3.06/4.95±3.5	7	1,2,3,4,5,7,8,9,11,12
Packiam,2012,USA	R	11	18	Minor	57.0/52.0	3/4	31.0/29.0	4.73±3.48/4.72±3.62	8	1,2,3,4,5,6,7,8,9,12,14
Rho,2019,Korea	R	40	169	Major and minor	Unclear	Unclear	Unclear	Unclear	5	9,10,11
Salloum,2016,France	R	16	80	Minor	Unclear	Unclear	Unclear	5.45±3.68/3.64±1.95	5	1,3,8,12
Spampinato,2014,Italy	R	25	25	Major	63.0/62.0	13/10	24.0/25.0	Unclear	7	1,2,3,5,6,7,8,9,11
Tranchart,2014,France	M	28	28	Minor	66.5/66.0	13/13	26.1/23.2	4.13±2.7/4.69±3.08	7	1,2,3,5,6,8,9,12,14
Troisi,2013,Belgium	R	40	223	Major and minor	64.6/54.1	27/98	Unclear	5.18±3.76/4.97±3.77	7	1,2,3,4,5,7,8,11,12,14
Tsung,2014,USA	M	57	114	Major and minor	58.4/58.7	24/47	Unclear	3.42±2.24/3.85±3.00	8	1,2,3,5,7,8,9,10,12,14
Wang,2019,China	R	92	48	Major	54.1/49.4	55/24	24.2/23.7	7.1±3.3/7.0±3.3	7	1,2,3,4,5,7,8,9,12,14
Wu,2014,China	R	38	41	Major and minor	60.9/54.1	32/28	Unclear	3.4±1.7/2.5±1.6	7	1,2,3,7,8,9,12,14
Yu,2014,Korea	R	13	17	Major and minor	50.4/52.5	7/9	Unclear	3.11±1.6/3.48±1.82	7	1,2,3,5,6,7,8,9,12,13,14

RLR = robotic-assisted liver resection; LLR = laparoscopic liver resection; P = prospectively collected data; R = retrospective non-matched comparative; M = retrospective matched comparative; Minor resections ≤ 2 segments; Major resections ≥ 3 segments; FI = France and Italy. Operation indications: 1 = operative time; 2 = estimated blood loss; 3 = overall surgical complications; 4 = minor surgical complications; 5 = major surgical complications; 6 = transfusion rate; 7 = length of hospital stay; 8 = conversion rate; 9 = mortality rate; 10 = R0 resection rate; 11 = R1 resection rate;12 = largest tumor size; 13 = total cost; 14 = malignant lesions rate.

### Quality judgments of studies

According to the Newcastle–Ottawa scale, the score obtained ranged from 5 to 8 (Table 1). Twenty-four articles were awarded 7 or 8 points, and considered as high-quality; Four studies were awarded 5 points and one study was awarded 6 points, which were considered as moderate quality.

### Analysis of intraoperative outcome indicators

#### Operative time

**Fig 2:**
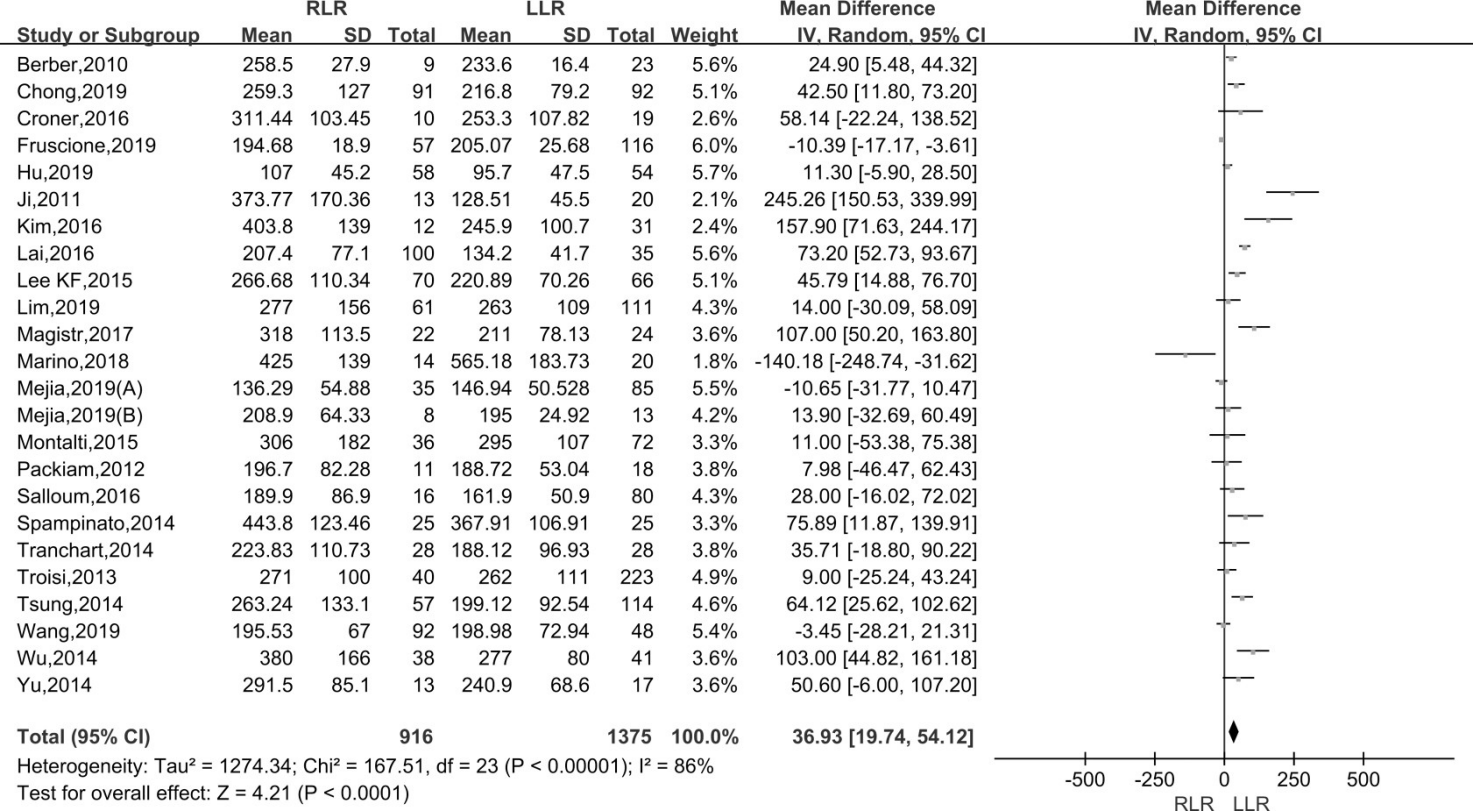
Forest plot of the meta-analysis on operative time.

Pooling data of twenty-four studies assessed the operative time in 2291 patients. As the I^2^ value showed the significant heterogeneity among the studies (I^2^ = 86%), the random-effect model was applied. The results showed that there was significantly longer operative time in the RLR than LLR group (WMD: 36.93; 95% CI, 19.74–54.12; P < 0.001) (Fig 2).

#### Estimated blood loss

**Fig 3:**
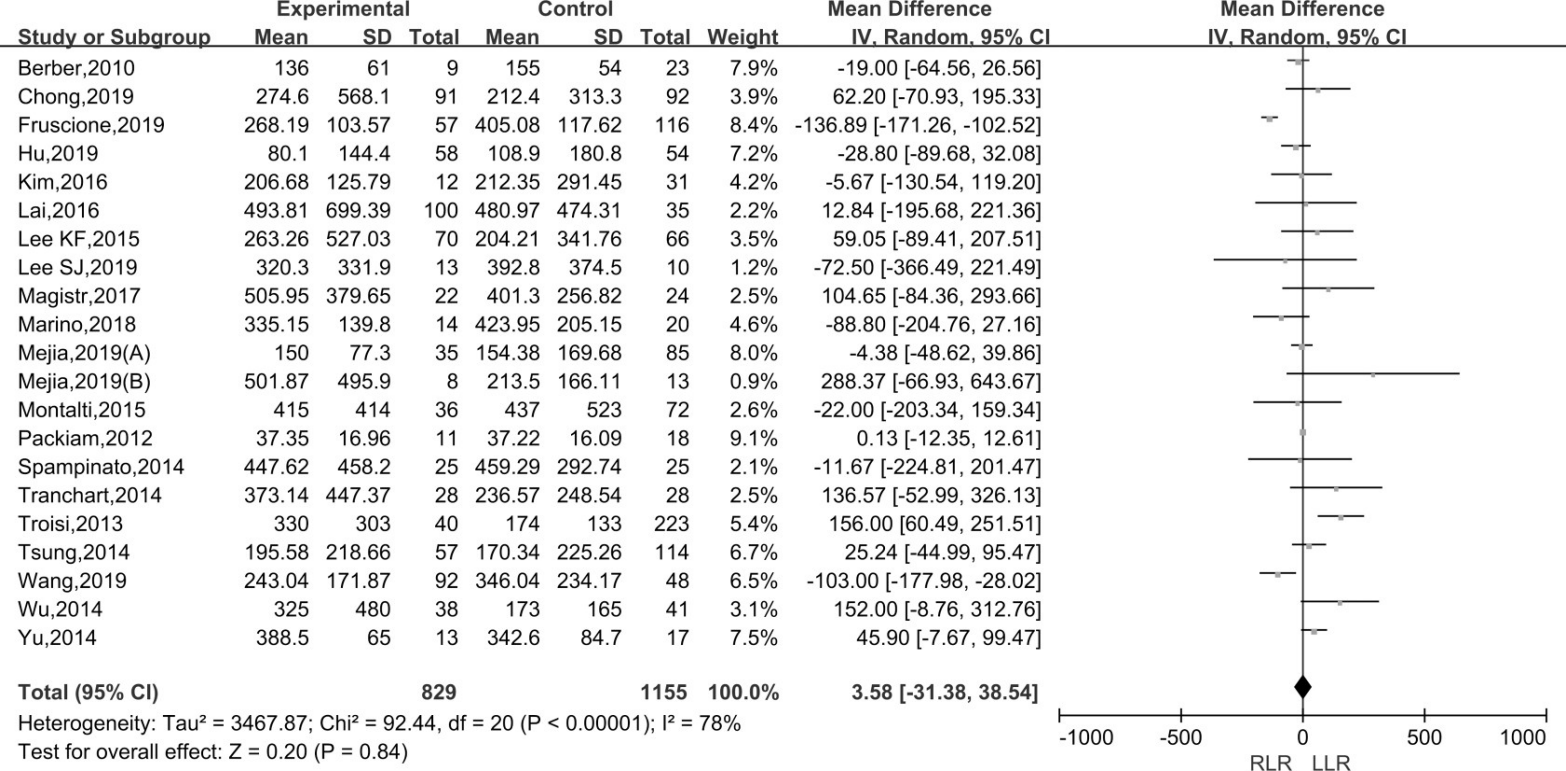
Forest plot of the meta-analysis on estimated blood loss.

Pooling data of twenty-one studies assessed the estimated blood loss in 1984 patients. As the I^2^ value showed the significant heterogeneity among the studies (I^2^ = 78%), the random-effect model was applied. The results showed that there was no statistically significant difference in the RLR versus LLR groups (WMD: 3.58; 95% CI, -31.38–38.54; P = 0.84) (Fig 3).

#### Transfusion rate

**Fig 4:**
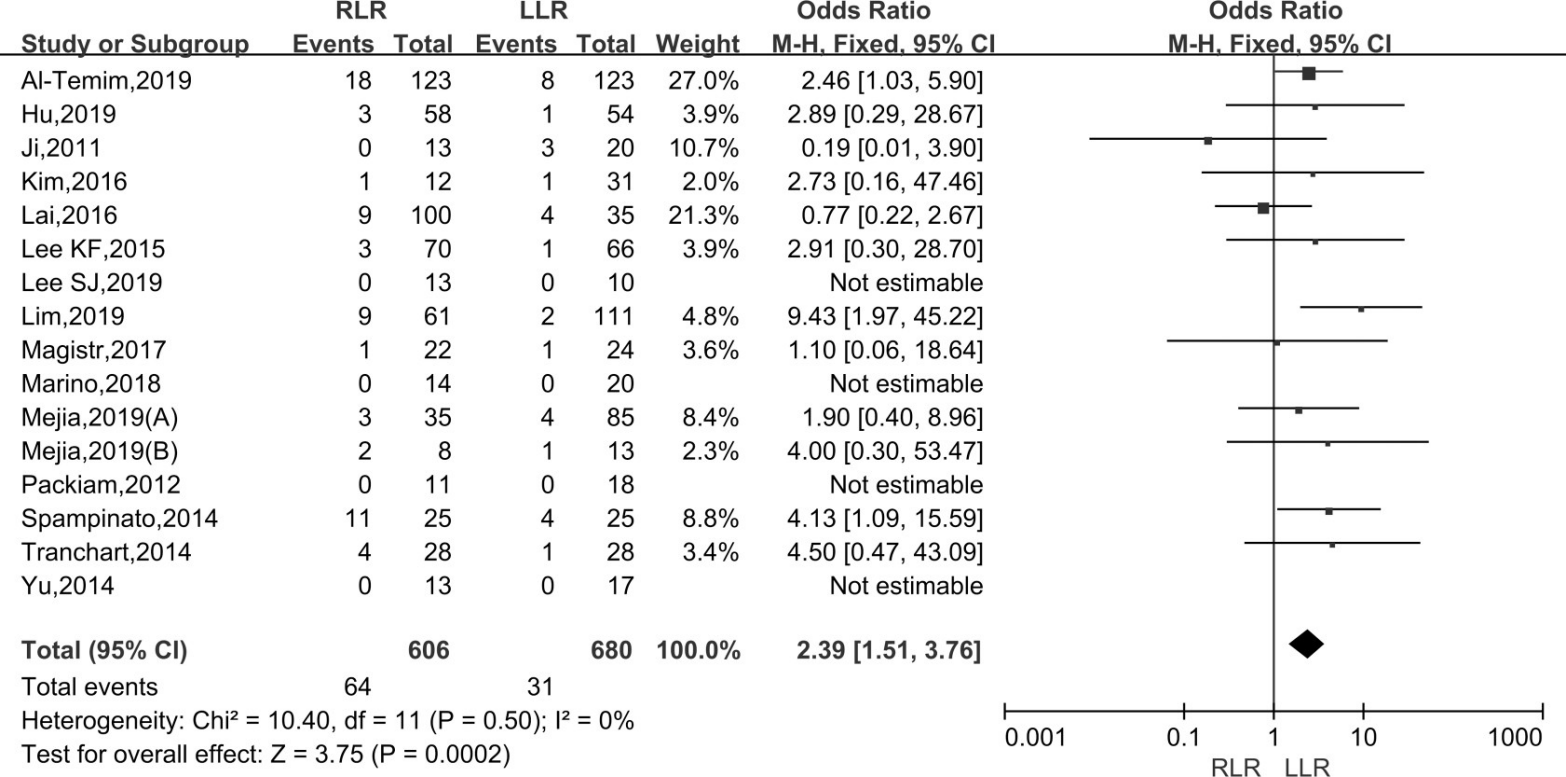
Forest plot of the meta-analysis on transfusion rate.

Pooling data of sixteen studies assessed the transfusion rate in 1286 patients. As the I^2^ value showed an absence of heterogeneity among the studies (I^2^ = 0%), the fixed-effect model was applied. The results showed that there was a significantly higher transfusion rate in the RLR than LLR group (WMD: 2.39; 95% CI, 1.51–3.76; P < 0.001) (Fig 4).

#### Conversion rate

**Fig 5:**
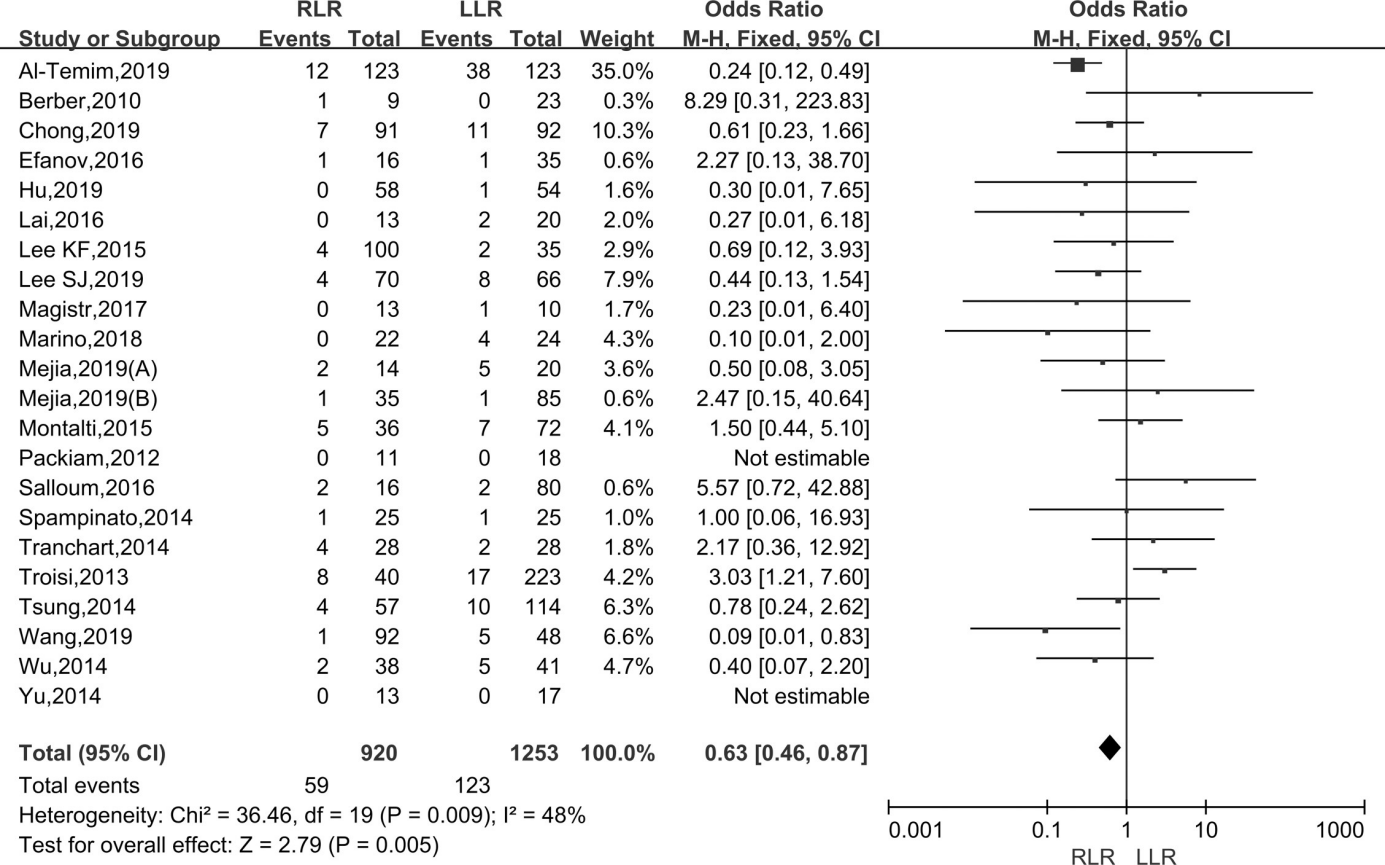
Forest plot of the meta-analysis on conversion rate.

Pooling data of twenty-two studies assessed the conversion rate in 2173 patients. Robotic surgery was converted to laparoscopic surgery or open surgery, while laparoscopic surgery was merely converted to open surgery. As the I^2^ value showed the insignificant heterogeneity among the studies (I^2^ = 48%), the fixed-effect model was applied. The results showed that there was a significantly lower conversion rate in the RLR than LLR group (OR: 0.63; 95% CI, 0.46–0.87; P = 0.005) (Fig 5).

### Analysis of postoperative outcome indicators

#### Surgical complications

Pooling data of twenty-five studies assessed overall surgical complications in 2448 patients. As the I^2^ value showed the insignificant heterogeneity among the studies (I^2^ = 9%), the fixed-effect model was applied. The results showed that there was no statistically significant difference in the RLR versus LLR groups (OR: 1.02; 95% CI, 0.81–1.28; P = 0.90) (S1 Fig).

According to Dindo-Calvien classification criteria for surgical complications [53], grade 1–2 was defined as minor complication, and grade 3–5 was defined as major complication. Overall surgical complications were further divided into minor and major complications. According to S2 and S3 Figs, there was no significant difference in minor (OR: 1.02; 95% CI, 0.72–1.43; P = 0.92) and major (OR: 0.94; 95% CI, 0.58–1.52; P = 0.79) complications between the two groups.

#### Length of hospital stay

Pooling data of twenty-three studies assessed the length of hospital stay in 2877 patients. As the I^2^ value showed the significant heterogeneity among the studies (I^2^ = 86%), the random-effect model was applied. The results showed that there was no statistically significant difference in the RLR versus LLR groups (WMD: -0.06; 95% CI, -0.47–0.34; P = 0.76) (S4 Fig).

#### Mortality rate

Pooling data of twenty-two studies assessed the mortality rate in 2713 patients. As the I^2^ value showed an absence of heterogeneity among the studies (I^2^ = 0%), the fixed-effect model was applied. The results showed that there was no statistically significant difference in the RLR versus LLR groups (OR: 0.56; 95% CI, 0.22–1.45; P = 0.23) (S5 Fig).

### Analysis of pathological outcome indicators

#### R0 and R1resection rate

No gross or microscopic tumor was evident along the transection surface is considered as R0 resection; microscopic tumor was evident is considered as R1 resection [54]. Nine studies reported the R0 resection rate and ten studies reported the R1 resection rate. Our pooled data showed that there were no significant difference in R0 (OR: 0.67; 95% CI, 0.37–1.19; P = 0.17) and R1 (OR: 0.79; 95% CI, 0.44–1.40; P = 0.42) resection rate between the two groups (S6 and S7 Figs).

#### Largest tumor size

**Fig 6:**
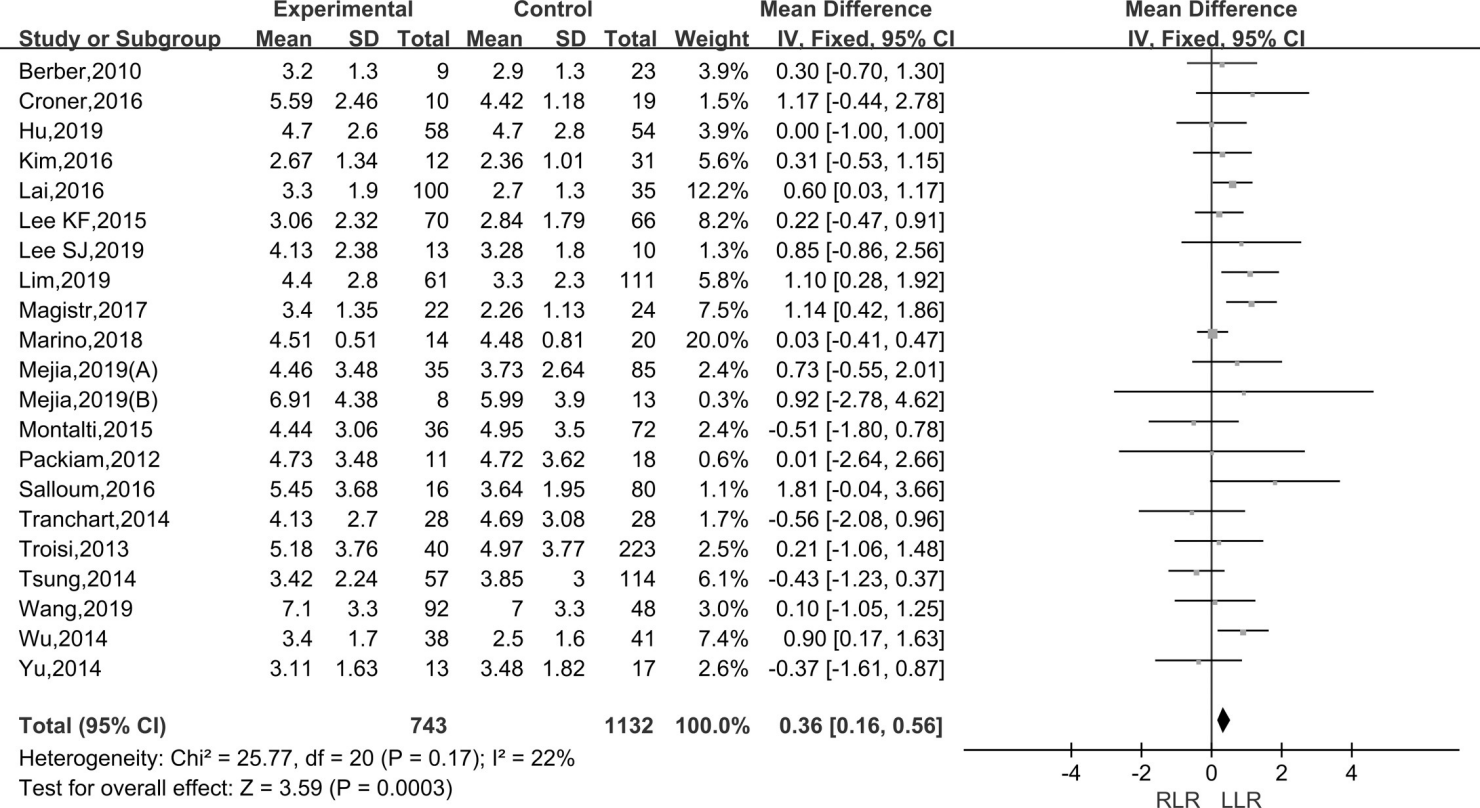
Forest plot of the meta-analysis on largest tumor size.

Pooling data of twenty-one studies assessed the baseline characteristics of patients about the largest tumor size in 1875 patients. As the I^2^ value showed the insignificant heterogeneity among the studies (I^2^ = 22%), the fixed-effect model was applied. The results showed that there was a significantly larger tumor size in the RLR than the LLR group (WMD: 0.36; 95% CI, 0.16–0.56; P < 0.001) (Fig 6).

#### Malignant lesions rate

**Fig 7:**
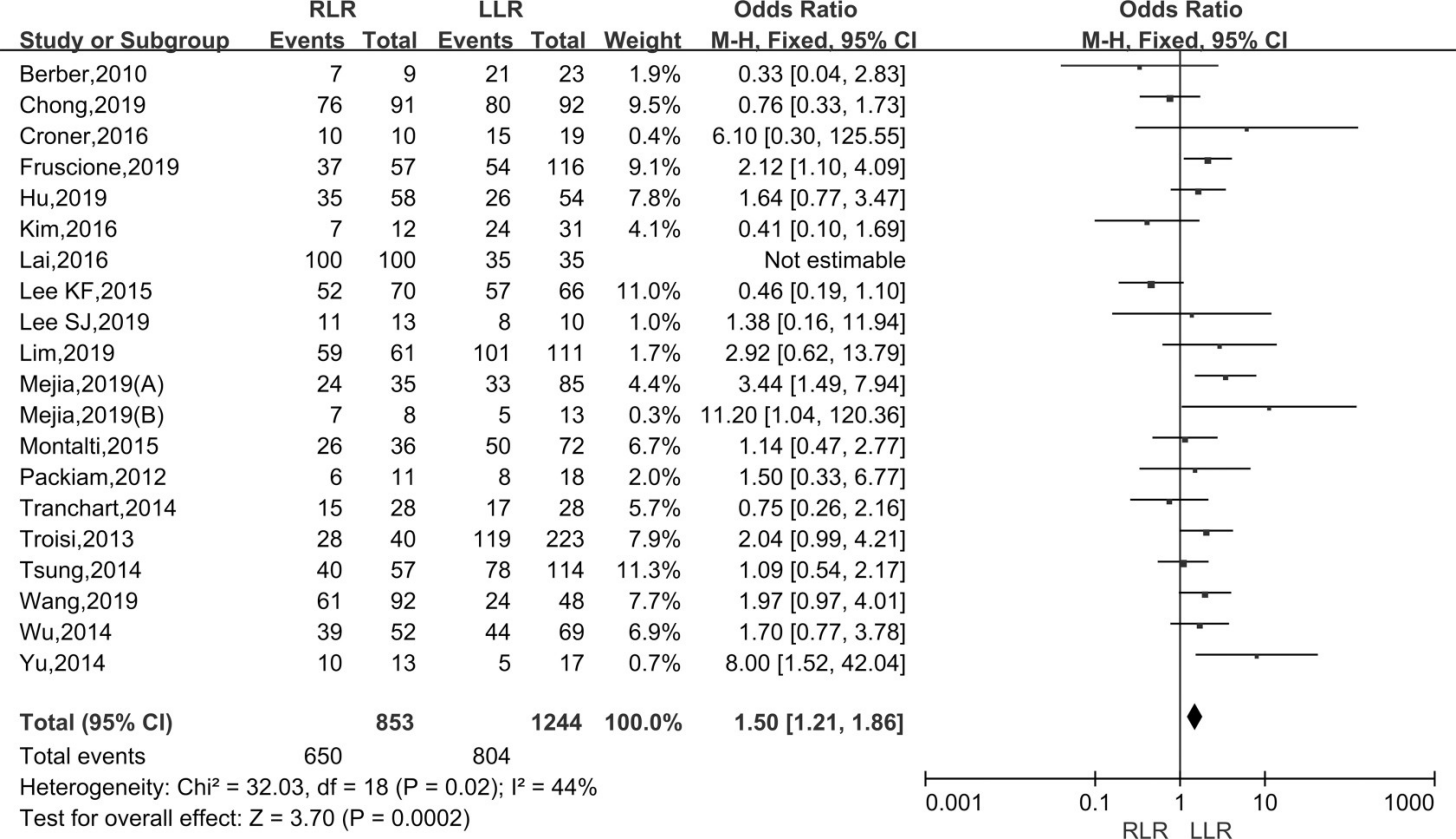
Forest plot of the meta-analysis on malignant lesions rate.

Pooling data of twenty studies assessed the baseline characteristics of patients about malignant lesions rate in 1703 patients. The malignant lesions included hepatocellular carcinoma, cholangiocarcinoma, gallbladder cancer, cystadenocarcinoma, liver metastasis, colorectal liver metastases, lung cancer liver metastasis, nasopharyngeal carcinoma liver metastasis. As the I^2^ value showed the insignificant heterogeneity among the studies (I^2^ = 44%), the fixed-effect model was applied. The results showed that there was a significantly higher malignant lesions rate in the RLR than the LLR group (WMD: 1.50; 95% CI, 1.21–1.86; P < 0.001) (Fig 7).

### Total cost

**Fig 8:**
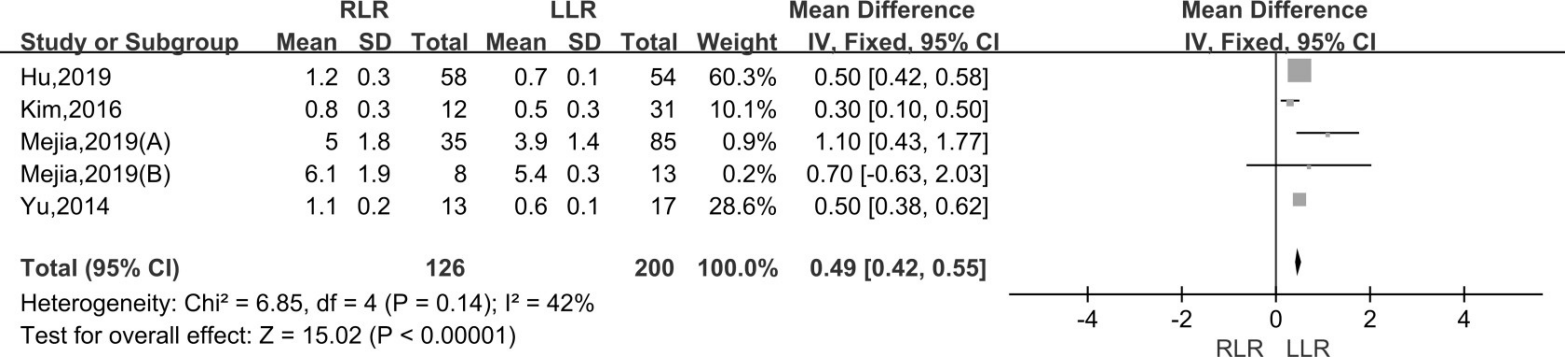
Forest plot of the meta-analysis on total cost.

Pooling data of five studies assessed total cost in 326 patients. As the I^2^ value showed the insignificant heterogeneity among the studies (I^2^ = 42%), the fixed-effect model was applied. The results showed that there was a significantly higher cost in the RLR versus LLR groups (WMD:0.49; 95% CI, 0.42–0.55; P < 0.001) (Fig 8).

### Subgroup analysis

#### Comparison on different country

In this subgroup analysis of the United States, outcomes demonstrated RLR was associated with higher transfusion rate (OR: 2.43; 95% CI, 1.17–5.03; P = 0.02) and lower R0 resection rate (OR: 0.45; 95% CI, 0.23–0.90; P = 0.02). In this subgroup analysis of China, outcomes demonstrated RLR was associated with longer operative time (WMD:57.36; 95% CI, 23.73–90.99; P < 0.001) and lower conversion rate (OR: 0.43; 95% CI, 0.24–0.77; P = 0.004). In this subgroup analysis of Italy, outcomes demonstrated RLR was associated with longer operative time (WMD: 66.09; 95% CI, 10.48–121.69; P = 0.02) and higher transfusion rate (OR: 3.25; 95% CI, 1.00–10.59; P = 0.05). In this subgroup analysis of Korea, outcomes demonstrated RLR was associated with higher total cost (WMD: 0.42; 95% CI, 0.22–0.61; P < 0.001). Just two studies came from France, there was only sufficient data to assess the operative time, overall surgical complications, and conversion rate. However, these outcomes all had no statistically significant difference in the RLR versus LLR groups (Table 2).

**Table 2:** Subgroup analysis of different country.

Outcomes	No. of studies	No. patients, RLR/LLR	Analysis model	OR/WMD [95% CI]	P value*	Study heterogeneity	
						I ^2^%	P value*
Operative time							
USA	6	177/369	RE	11.72 [-9.75, 33.19]	0.28	80	<0.001
China	7	462/356	RE	57.36 [23.73, 90.99]	**<0.001**	89	<0.001
Italy	3	83/121	RE	66.09 [10.48, 121.69]	**0.02**	59	0.09
Korea	2	25/48	RE	99.11 [-5.56, 203.78]	0.06	76	0.04
France	2	44/108	FE	31.04 [-3.20, 65.29]	0.08	0	0.83
Estimated blood loss							
USA	6	177/369	RE	-21.34 [-79.50, 36.83]	0.47	91	<0.001
China	6	449/336	RE	5.21 [-66.55, 76.96]	0.89	58	0.04
Italy	3	83/121	FE	24.91 [-86.60, 136.43]	0.66	0	0.59
Korea	3	38/58	FE	34.87 [-13.68, 83.43]	0.16	0	0.58
Transfusion rate							
USA	4	177/239	FE	2.43 [1.17, 5.03]	**0.02**	0	0.89
China	3	183/121	FE	0.83 [0.32, 2.15]	0.70	5	0.35
Italy	2	47/49	FE	3.25 [1.00, 10.59]	**0.05**	0	0.41
Conversion rate							
USA	5	235/363	RE	0.76 [0.21, 2.80]	0.68	63	0.05
China	7	462/356	FE	0.43 [0.24, 0.77]	**0.004**	0	0.84
Italy	3	83/121	FE	0.81 [0.30, 2.14]	0.66	30	0.24
France	2	44/108	FE	3.03 [0.76, 12.04]	0.12	0	0.49
Overall surgical complications							
USA	7	300/492	FE	0.93 [0.65, 1.34]	0.71	32	0.18
China	6	404/302	FE	1.23 [0.74, 2.05]	0.43	0	0.57
Italy	3	83/121	RE	0.74 [0.39, 1.41]	0.41	80	0.008
Korea							
France	3	38/58	FE	0.84 [0.25, 2.86]	0.78	0	0.59
	2	44/108	FE	0.87 [0.31, 2.45]	0.80	0	0.83
Minor surgical complications							
USA	4	111/232	FE	0.94 [0.49, 1.81]	0.85	21	0.29
China	3	196/160	FE	1.14 [0.52, 2.49]	0.74	0	0.94
Italy	3	83/121	RE	0.67 [0.18, 2.46]	0.55	67	0.05
Major surgical complications							
USA	5	168/346	FE	0.64 [0.25, 1.66]	0.36	0	0.71
China	3	196/160	FE	4.03 [0.48, 33.75]	0.20	0	0.49
Italy	3	83/121	FE	0.91 [0.34, 2.42]	0.85	0	0.44
Korea	2	26/27	FE	0.67 [0.10, 4.53]	0.68	6	0.30
Length of hospital stay							
USA	6	372/866	RE	-0.33 [-1.06, 0.40]	0.37	96	<0.001
China	6	449/336	FE	0.04 [-0.31, 0.40]	0.80	0	0.89
Italy	3	83/121	RE	0.19 [-1.35, 1.72]	0.81	64	0.06
Korea	3	95/114	RE	0.07 [-1.11, 1.26]	0.90	61	0.08
Mortality rate							
USA	6	438/873	FE	0.43 [0.11, 1.59]	0.20	0	0.41
Italy	3	83/121	FE	1.36 [0.20, 9.16]	0.75	37	0.21
R0 resection rate							
USA	3	100/212	FE	0.45 [0.23, 0.90]	**0.02**	0	0.59
China	3	249/181	FE	1.82 [0.46, 7.09]	0.39	0	0.61
R1 resection rate							
Italy	3	83/121	FE	0.57 [0.21, 1.57]	0.28	3	0.36
Total cost							
USA	3	247/618	RE	0.35 [-0.95, 1.66]	0.60	91	<0.001
Korea	2	25/48	RE	0.42 [0.22, 0.61]	**<0.001**	65	0.09

RLR = robotic-assisted liver resection; LLR = laparoscopic liver resection; OR = odds ratio; WMD = weighted mean difference; CI = confidence interval; Minor surgical complications = Clavien-Dindo grades (1–2); Major surgical complications = Clavien-Dindo grades (3–5); FE = fixed-effect model; RE = random-effect model.

* Statistically significant results were shown in bold.

#### Comparison in minor and major hepatectomy

In this subgroup analysis, resection extent ≤ 2 segments were considered “minor hepatectomy”, resection extent ≥ 3 segments were considered “major hepatectomy”. Eleven and six studies reported minor hepatectomy and major hepatectomy, respectively. It was worth noting that Lee et al. [48] not only compared all patients in RLR with LLR, but compared the patients of minor hepatectomies between RLR with LLR. In addition, three different comparisons were performed in the study by Tsung et al. [43] Firstly, all patients in RLR were compared with all case-matched patients in LLR. After that, 108 patients who underwent minor hepatectomies were compared between RLR and LLR. Meanwhile, 63 patients who underwent major hepatectomies were compared between RLR and LLR. Others were mixed liver resection (both major and minor), so we failed to perform a meta-analysis.

In this subgroup analysis of minor hepatectomy, outcomes demonstrated RLR was associated with longer operative time (WMD: 36.00; 95% CI, 12.59–59.41; P = 0.003), longer length of stay (WMD: 0.51; 95% CI, 0.02–1.01; P = 0.04) and higher total cost (WMD: 0.48; 95% CI, 0.25–0.72; P < 0.001) (Table 3). In this subgroup analysis of major hepatectomy, outcomes demonstrated RLR was associated with lower estimated blood loss (WMD: -122.43; 95% CI, -151.78 - -93.08; P **<** 0.001) (Table 3).

**Table 3:** Subgroup analysis of minor and major hepatectomy.

Outcomes	No. of studies	No. patients, RLR/LLR	Analysis model	OR/WMD [95% CI]	P value*	Study heterogeneity	
						I ^2^%	P value*
Operative time							
Minor	11	289/511	RE	36.00 [12.59, 59.41]	**0.003**	83	<0.001
Major	6	217/264	RE	7.60 [-21.55, 36.75]	0.61	73	0.002
Estimated blood loss							
Minor	9	263/412	RE	32.16 [-31.47, 95.78]	0.32	92	<0.001
Major	6	217/264	FE	-122.4 [-151.8, -93.1]	**<0.001**	33	0.19
Transfusion rate							
Minor	7	218/317	FE	2.29 [0.93, 5.65]	0.07	0	0.96
Major	4	68/100	RE	1.85 [0.34, 10.03]	0.48	54	0.11
Conversion rate							
Minor	9	267/3461	FE	1.25 [0.64, 2.42]	0.52	10	0.36
Major	4	152/135	FE	0.53 [0.22, 1.26]	0.15	24	0.27
Overall surgical complications							
Minor	10	231/457	FE	1.13 [0.71, 1.81]	0.60	0	0.53
Major	6	217/264	FE	0.71 [0.45, 1.12]	0.15	0	0.51
Minor surgical complications							
Minor	7	194/323	FE	1.24 [0.69, 2.23]	0.47	0	0.42
Major	4	203/224	FE	0.75 [0.45, 1.25]	0.27	0	0.90
Major surgical complications							
Minor	7	194/323	FE	1.05 [0.43, 2.60]	0.91	0	0.92
Major	5	203/244	FE	0.70 [0.30, 1.64]	0.41	0	0.52
Length of hospital stay							
Minor	9	247/408	RE	0.51 [0.02, 1.01]	**0.04**	64	0.005
Major	6	217/264	RE	0.18 [-0.75, 1.11]	0.70	75	0.001
Mortality rate							
Minor	9	264/408	FE	0.97 [0.23, 4.07]	0.97	0	0.60
Major	5	93/125	FE	0.34 [0.05, 2.16]	0.25	0	1.00
R0 resection rate							
Minor	4	139/230	FE	0.54 [0.23, 1.25]	0.15	0	0.89
Major	3	68/147	FE	0.45 [0.18, 1.14]	0.09	26	0.26
Total cost							
Minor	3	105/170	RE	0.48 [0.25, 0.72]	**<0.001**	70	0.04

RLR = robotic-assisted liver resective; LLR = laparoscopic liver resection; OR = odds ratio; WMD = weighted mean difference; CI = confidence interval; Minor surgical complications = Clavien-Dindo grades (1–2); Major surgical complications = Clavien-Dindo grades (3–5); Minor hepatectomy≤2 segments; Major hepatectomy≥3 segments; FE = fixed-effect model; RE = random-effect model.

* Statistically significant results were shown in bold.

### Publication bias and sensitivity analysis

Egger's test results, coupled with funnel plots (S8 Fig), indicated that there was significant publication bias about the operative time (P = 0.001). However, Begg test had the opposite result (P = 0.189). The risk of publication bias was also assessed for other outcomes and showed symmetry, suggesting that publication bias was not large and was unlikely to drive conclusions.

Twenty retrospective non-matched comparatives studies and four retrospective matched comparative studies were included in the sensitivity analysis. The degree of between-study heterogeneity decreased slightly for operative time and largest tumor size but increased slightly for estimated blood loss, conversion rate, overall surgical complications, length of stay, and malignant lesions rate. The heterogeneity remained statistically significant for operative time, estimated blood loss and length of stay. The results of sensitivity analysis were not altered after excluding the studies of prospectively collected data (Table 4).

**Table 4:** Sensitivity analysis of retrospective non-matched comparative and retrospective matched comparative studies.

Outcomes	No. of studies	No.patients,RLR/LLR	Analysis model	OR/WMD [95% CI]	P value*	Study heterogeneity	
						I^2^,%	P value*
Operative time	19	645/1095	RE	35.08 [15.90,54.25]	**<0.001**	84	<0.001
Estimated blood loss	18	629/1005	RE	4.40 [-35.70, 44.51]	0.83	81	<0.001
Conversion rate	19	720/1103	FE	0.73 [0.39, 0.98]	0.04	49	0.005
Transfusion rate	14	445/534	FE	2.40 [1.42, 4.04]	**0.001**	0	0.89
Overall surgical complications	20	723/1174	FE	0.96 [0.74, 1.25]	0.77	14	0.29
Minor surgical complications	12	380/682	FE	0.86 [0.57, 1.28]	0.45	0	0.46
Major surgical complications	12	450/813	FE	0.23 [0.01, 5.21]	0.54	0	0.94
Length of hospital stay	19	833/1525	RE	-0.07 [-0.51, 0.38]	0.77	88	<0.001
Mortality rate	19	899/1478	FE	0.56 [0.21, 1.51]	0.25	0	0.52
R0 resection rate	7	211/455	FE	0.53 [0.28, 1.01]	0.05	0	0.63
R1 resection rate	11	330/704	FE	0.83 [0.40, 1.74]	0.63	0	0.65
Largest tumor size	17	563/944	FE	0.26 [0.03, 0.48]	**0.03**	21	0.21
Malignant lesions rate	15	582/964	FE	1.56 [1.24, 1.96]	**<0.001**	46	0.03
Total cost	5	126/200	FE	0.49 [0.42, 0.55]	**<0.001**	42	0.14

RLR = robotic-assisted liver resection; LLR = laparoscopic liver resection; OR = odds ratio; WMD = weighted mean difference; CI = confidence interval; Minor surgical complications = Clavien-Dindo grades (1–2); Major surgical complications = Clavien-Dindo grades (3–5); FE = fixed-effect model; RE = random-effect model.

* Statistically significant results were shown in bold.

## Discussion

### Summary of results

The largest number of published articles and cases were included in this meta-analysis. The results showed no differences in estimated blood loss, surgical complications, length of hospital stay, mortality rate, R0 and R1 resection rate between the RLR and LLR group. It was worth noting that a significant decrease in conversion rate, and a significant increase in operative time, transfusion rate and total cost was observed in the RLR group when compared to the LLR group. These results were not completely consistent with other relevant meta-analyses [11–14]. We thought that our meta-analysis presented more reliable results due to more high-quality studies included and the quality of the included studies assesed rigorously by NOS. In addition, we revealed for the first time that RLR has certain advantages in major hepatectomy compared with LLR, and surgeons were more likely to use the robot-assisted system to perform more complex liver operations, such as large tumor size and malignant lesions.

### Explanation of results

As to the operative time, the results revealed that longer operative time was observed in the RLR than LLR group [55]. However, with the accumulation of the surgeon's experience and the improvement of robot-assisted technology, the operation time will be greatly shortened. Tsung et al. [43] showed that as the number of robot-assisted surgical cases accumulated, the terminal phase was statistically significant shorter when compared to the initial phase. Notably, the 2018 international consensus statement on RLR indicated that, although the learning curve of the robot-assisted system is improved significantly, LLR still takes less time [55]. The advantage of RLR is not apparent, and it may be because the largest tumor size is larger, and the malignant lesions rate is higher in the RLR than LLR group, which makes the operation more difficult. Besides, the time took to assemble the robotic system before surgery accounts for a large proportion of the total surgery time. Fortunately, the Da Vinci robotic system has come out for about 20 years now. Compared with the first generation of the Da Vinci robot, the latest generation of robots has greatly simplified the assembly and further reduces the operation time [56].

As to the estimated blood loss and transfusion rate, interestingly, despite equivalent estimated blood loss in the RLR and LLR group, there was a significantly higher transfusion rate after RLR. The result cannot be readily explained and could possibly reflect different policies on operative management in different regions. But, all of the included studies did not report the principles of transfusion, therefore, we cannot investigate whether the principles of transfusion in different studies were similar or not. To our knowledge, robotic surgery can control bleeding during the operation more easily because of the three-dimensional optics and the seven degrees of freedom, but this advantage was not reflected in our meta-analysis. The largest tumor size of the RLR group was larger than the LLR group, which might cause with more blood loss. Subgroup analysis of major hepatectomy showed there was lower estimated blood loss in the RLR group than LLR group. Recently, an interesting finding was that blood transfusion increased surgical complication rates and decreased disease-free survival rates after hepatectomy of tumors patients [57]. Besides, blood transfusions increase the risk of postoperative recurrence and destroy anticancer immune response. Hence, decreasing the transfusion rate is an important part of the robot-assisted surgical development as well as an urgent need to improve.

As to conversion rate, our results showed that there was a significantly lower conversion rate in the RLR than LLR group. This may be because the robot-assisted system provides a larger and clearer 3‐dimensional field of vision so that surgeons can clearly identify anatomical structures, and the flexible “endo‐wrist”allows more precise dissection and the ease in suturing.

Another difference we found was that the total cost was significantly higher in the RLR group. To our knowledge, the robot-assisted system not only needs the higher capital cost of equipment and annual maintenance fee, but need to add $ 500 laparoscopic equipment [47]. The application and popularization of robot-assisted surgery system were restricted by the high cost of purchase, maintenance and operation. In addition, the high cost that was not covered by medical insurance also has limited the further development of robot-assisted surgery to a certain extent [37]. Therefore, reducing cost and introducing insurance coverage is extremely important measures to promote the widely used of robot-assisted surgery in clinical practice, especially in developing countries with the backward economy.

For many surgeons, minimally invasive major hepatectomy remains a challenge. In anatomical standard major hepatectomy, LLR is a viable alternative to open liver resection when vascular or biliary reconstruction is not required [58]. However, vascular or biliary reconstruction is technically easier with robot-assisted system [59]. Our subgroup analysis showed that RLR has certain advantages in major hepatectomy compared with LLR, which had no statistically significant difference in clinical results except for lower estimated blood loss. Unfortunately, only one study of Mejia et al. evaluated the total cost and found that there no significant difference in the total cost in major hepatectomy between the RLR and LLR group.

The heterogeneity of the results of operative time, estimated blood loss, conversion rate and length of hospital stay was significant, and the reasons may arise from the following aspects. (1) In different studies, the surgeons came from different research institutions, and their proficiency in the use of Da Vinci was very different. Our subgroup analysis proved this, for example there was no statistically significant heterogeneity about estimated blood loss in Italy and Korea. (2) Liver resection extent and type varied in the included studies. Our subgroup analysis showed that there was no statistically significant heterogeneity in estimated blood loss in major and minor liver resection. (3) The technical differences in terms of type or version of the robot-assisted system, intraoperative ultrasound methodology, and trocars positioning may also cause the heterogeneity. However, there is currently no sufficient data to evaluate these factors. In addition, various definitions of operative time and length of hospital stay might also be the potential source of heterogeneity. Therefore, future studies should have a uniform standard of the operative time and length of hospital stay.

### Study limitations

The current meta-analysis has the following several limitations. First, both the RLR and LLR were investigated in all kinds of liver tumors (HCC, cholangiocarcinoma, hemangioma, and so on), and there may be specific clinicopathologic impacts among these cases, which can cause the reporting bias, but we failed to acquire enough information to further assess these impacts separately. Second, all of the studies we included were not randomized controlled trials, and blind methods were not implemented. Third, some high-quality, large-sample studies were not included due to only reporting RLR or LLR data. Fourth, some important parameters, such as long-term follow-up results, in our included studies were inadequate to be evaluated. Therefore, we need more high-quality, large-sample randomized controlled studies to evaluate the advantages and disadvantages of robot-assisted surgical systems.

## Conclusion

Our meta-analysis revealed that RLR was associated with longer operative time, lower conversion rate, higher transfusion rate and total cost, and RLR has certain advantages in major hepatectomy compared with LLR. In addition, surgeons were more likely to use robot-assisted surgery to perform more complex liver operations, such as large tumor size and malignant lesions. Although we have adopted a rigorous methodology, due to the limitations of the included study, we were unable to draw clear conclusions. Future large-sample size, well-performed RCTs with long-term follow-up are warranted to resolve these disputes. In conclusion, current evidence did not demonstrate that the RAR is safer or more effective than LLR for liver diseases, which revealed that RAR was a developing procedure instead of replacing LLR at once.

## Supporting information

S1 Checklist PRISMA 2009 checklist. (DOC)

S1 Fig Forest plot of the meta-analysis on overall surgical complications. (TIF)

S2 Fig Forest plot of the meta-analysis on minor surgical complications. (TIF)

S3 Fig Forest plot of the meta-analysis on major surgical complications. (TIF)

S4 Fig Forest plot of the meta-analysis on length of hospital stay. (TIF)

S5 Fig Forest plot of the meta-analysis on mortality rate. (TIF)

S6 Fig Forest plot of the meta-analysis on R0 resection rate. (TIF)

S7 Fig Forest plot of the meta-analysis on R1 resection rate. (TIF)

S8 Fig Funnel plot of overall surgical complications in all included studies. (TIF)

S1 File Details of Search Strategy Used for Embase. (DOCX)
