# Supplementary figures and images for: Comparative clinical outcomes of robot-assisted liver resection versus laparoscopic liver resection: A meta-analysis
Source: PLoS One. 2020 Oct 13;15(10):e0240593. doi: 10.1371/journal.pone.0240593 (PMC7553328; doi:10.1371/journal.pone.0240593)

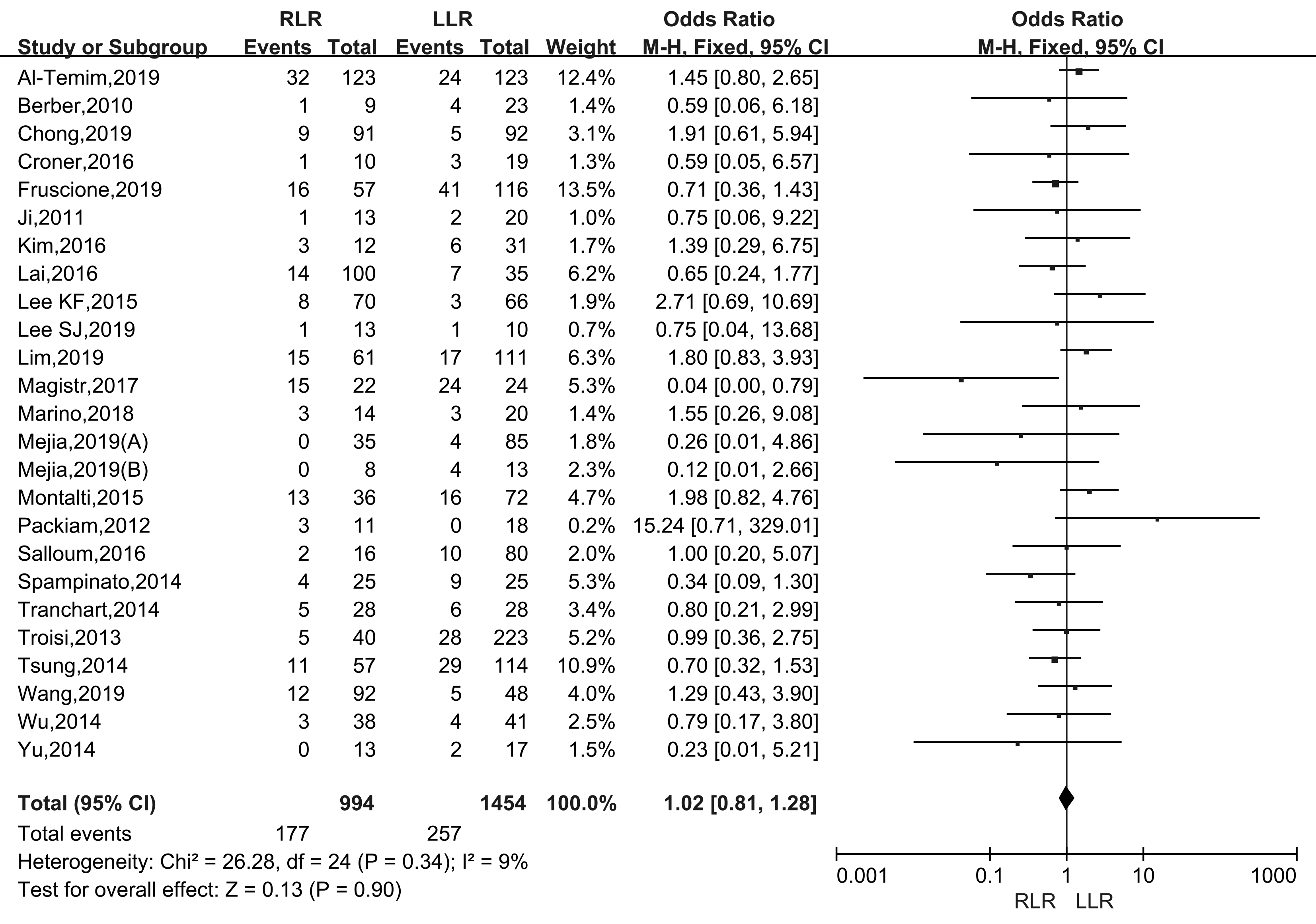

Supplement: S1 Fig — (TIF) [file pone.0240593.s002.tif]

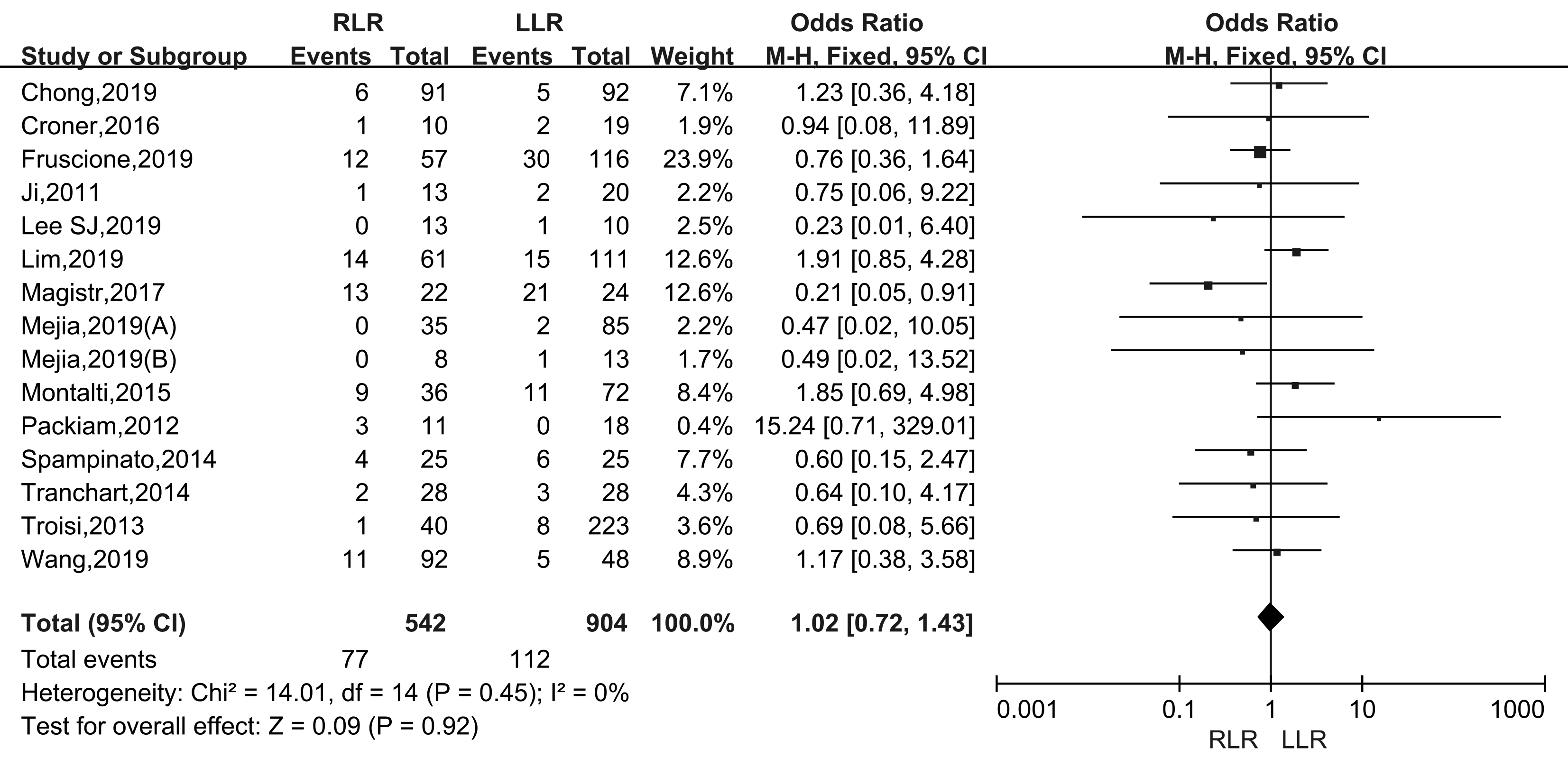

Supplement: S2 Fig — (TIF) [file pone.0240593.s003.tif]

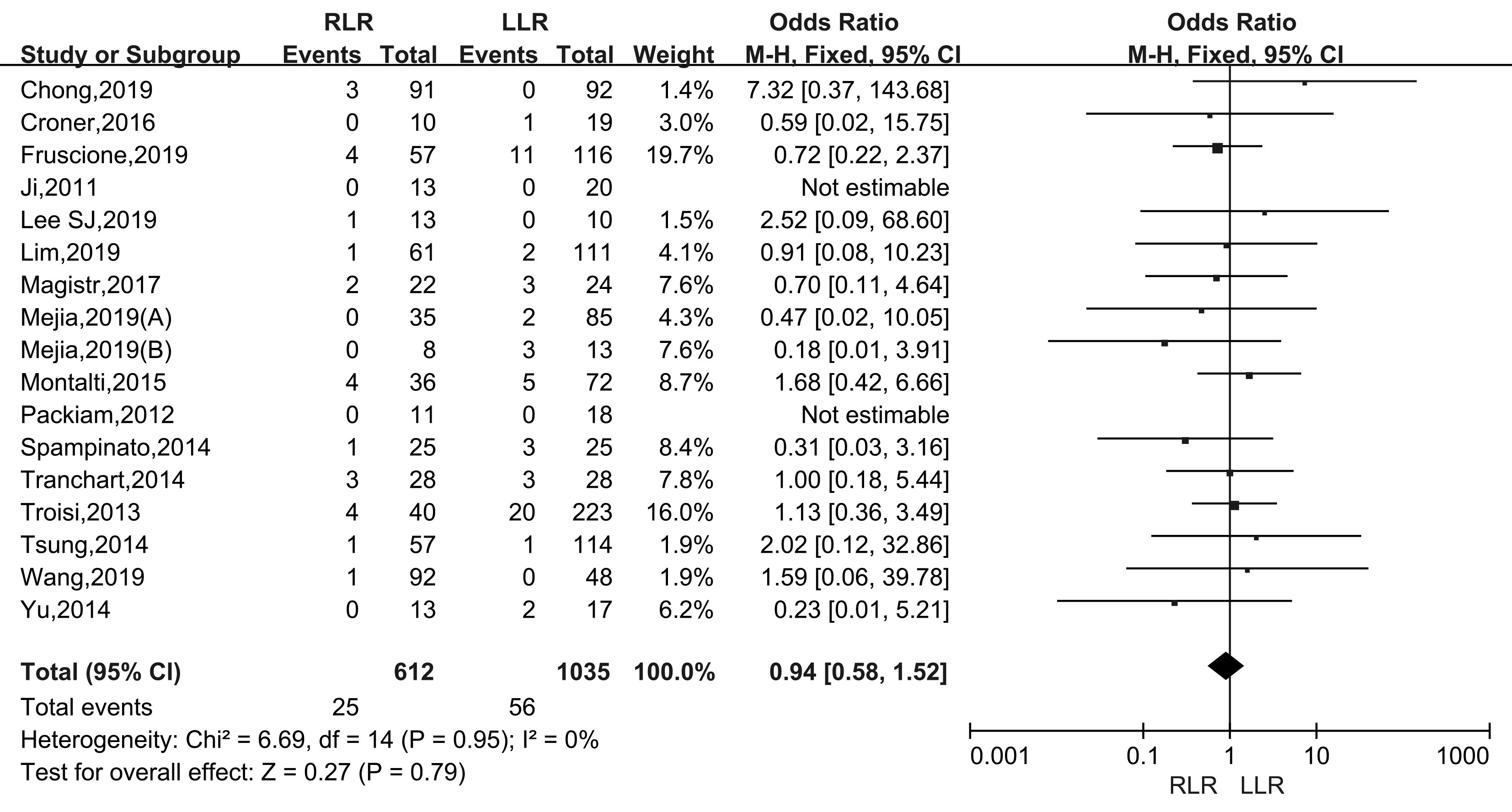

Supplement: S3 Fig — (TIF) [file pone.0240593.s004.tif]

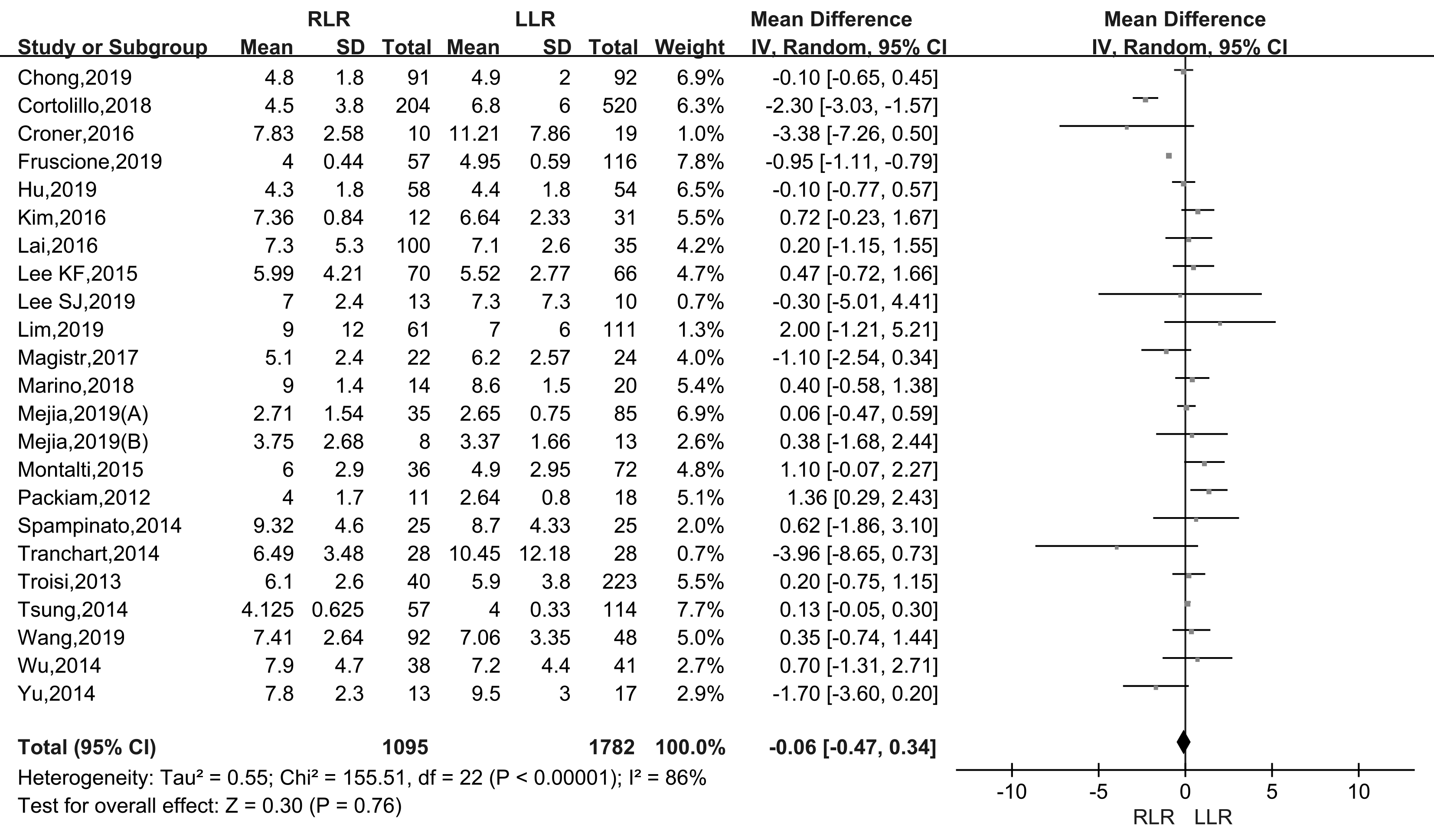

Supplement: S4 Fig — (TIF) [file pone.0240593.s005.tif]

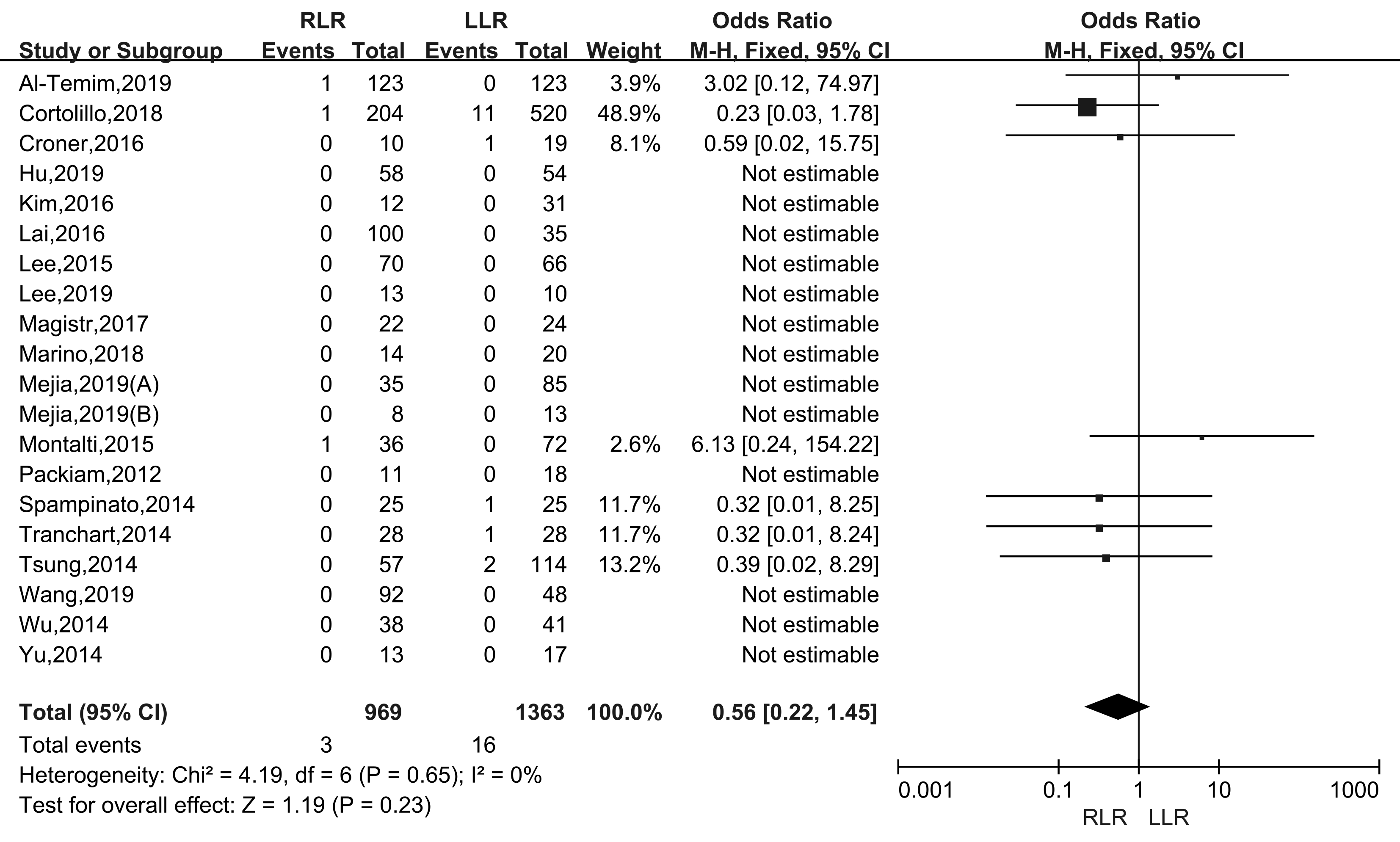

Supplement: S5 Fig — (TIF) [file pone.0240593.s006.tif]

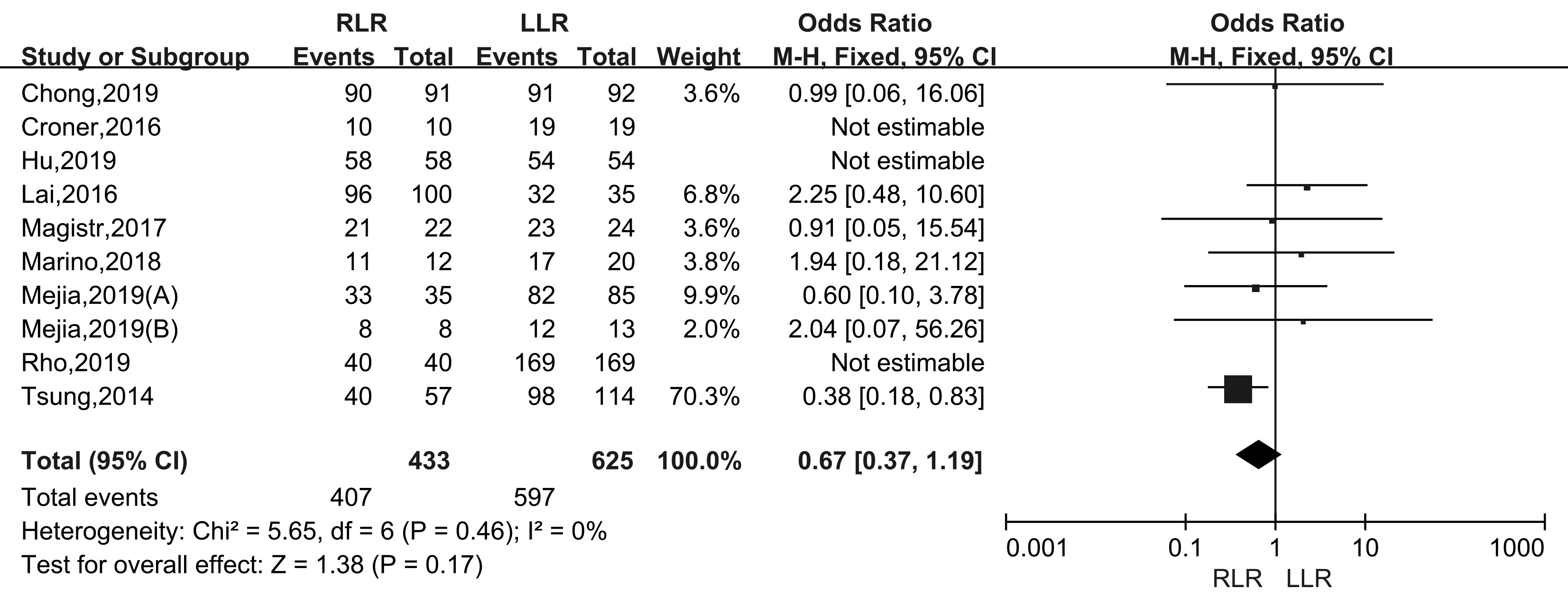

Supplement: S6 Fig — (TIF) [file pone.0240593.s007.tif]

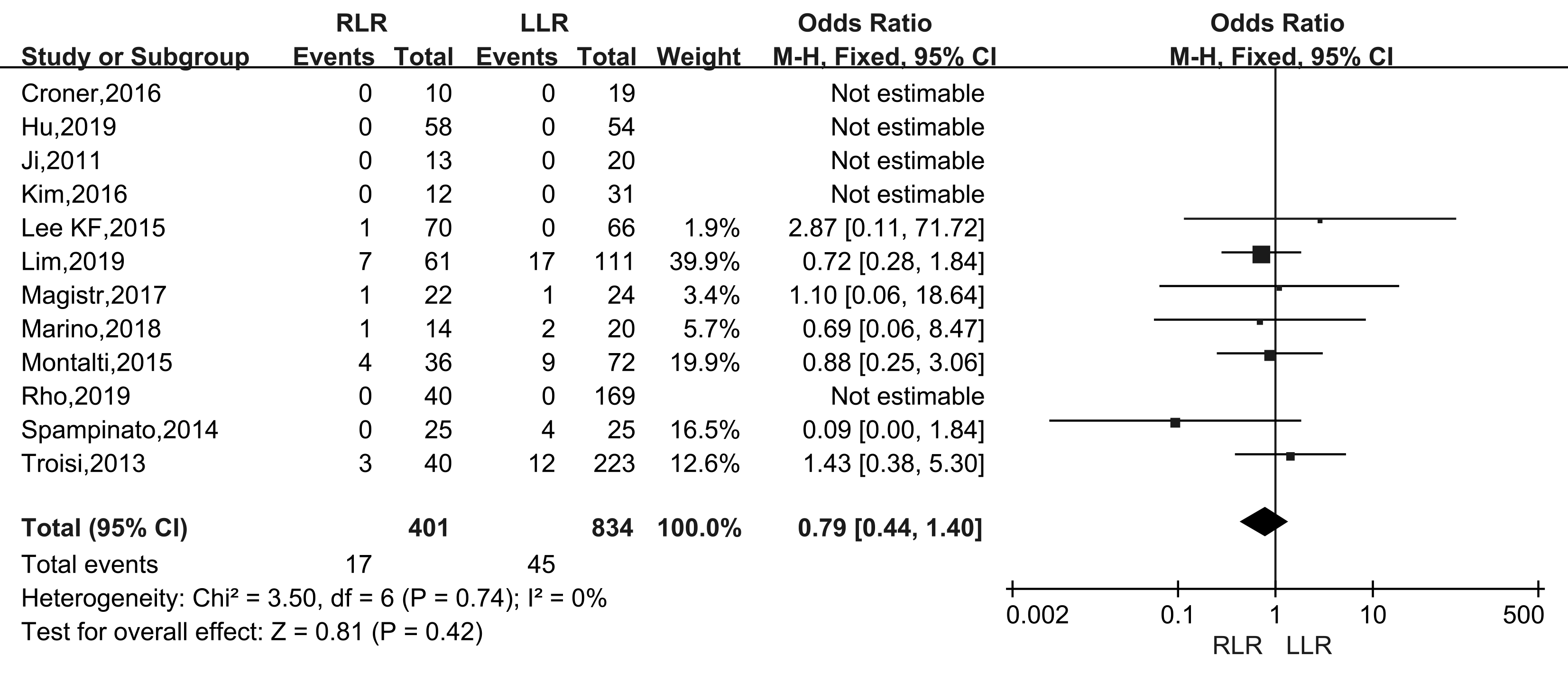

Supplement: S7 Fig — (TIF) [file pone.0240593.s008.tif]

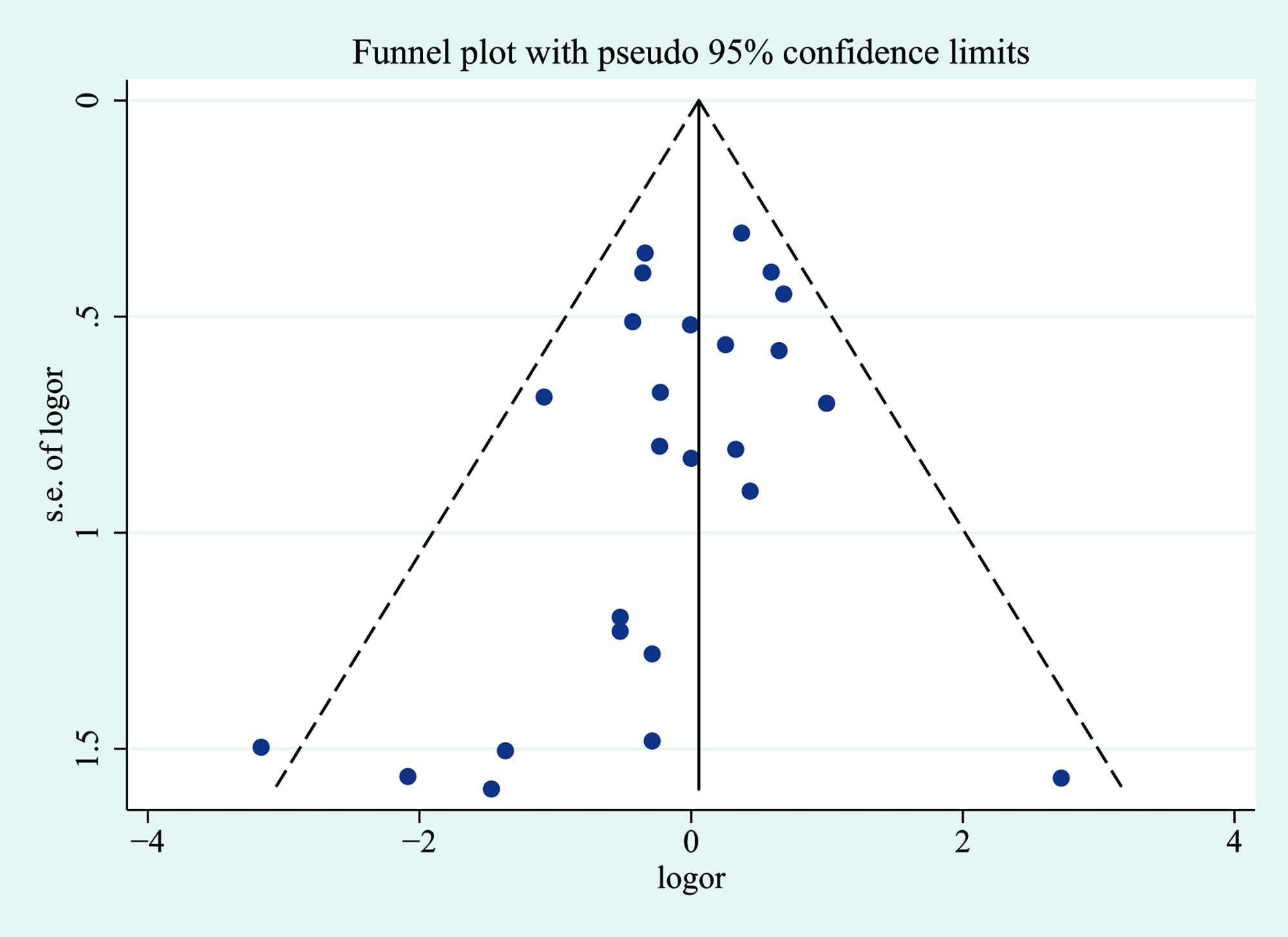

Supplement: S8 Fig — (TIF) [file pone.0240593.s009.tif]
