# Supplementary material for: Comparative clinical outcomes of robot-assisted liver resection versus laparoscopic liver resection: A meta-analysis
Source: PLoS One. 2020 Oct 13;15(10):e0240593. doi: 10.1371/journal.pone.0240593 (PMC7553328; doi:10.1371/journal.pone.0240593)
Supplement: S1 File — (DOCX) [file pone.0240593.s010.docx]

**Details of Search Strategy Used for Embase**

#1 'robotics'/exp

#2 robot*:ab,ti OR 'da vinci':ab,ti OR computer‐assisted:ab,ti OR telerobotics:ab,ti

#3 #1 OR #2

#4 'laparoscopy'/exp

#5 laparoscop*:ab,ti OR coelioscop*:ab,ti OR celioscop*:ab,ti OR peritoneoscop*:ab,ti

#6 #4 OR #5

#7 'hepatectomy'/exp

#8 'hepatic resection':ab,ti OR 'liver resection':ab,ti OR 'liver surgery':ab,ti

#9 #7 OR #8

#10 #3 AND #6 AND #9
